# Determinants and consequences of short birth interval in rural Bangladesh: a cross-sectional study

**DOI:** 10.1186/s12884-014-0427-6

**Published:** 2014-12-24

**Authors:** Hendrik CC de Jonge, Kishwar Azad, Nadine Seward, Abdul Kuddus, Sanjit Shaha, James Beard, Anthony Costello, Tanja AJ Houweling, Ed Fottrell

**Affiliations:** Institute for Global Health, University College London, London, United Kingdom; Department of Public Health, Erasmus University Medical Centre, Rotterdam, the Netherlands; Perinatal Care Project, Diabetic Association of Bangladesh, Dhaka, Bangladesh

**Keywords:** Birth intervals, Bangladesh, Perinatal mortality, Socioeconomic factors, Population surveillance

## Abstract

**Background:**

Short birth intervals are known to have negative effects on pregnancy outcomes. We analysed data from a large population surveillance system in rural Bangladesh to identify predictors of short birth interval and determine consequences of short intervals on pregnancy outcomes.

**Methods:**

The study was conducted in three districts of Bangladesh – Bogra, Moulavibazar and Faridpur (population 282,643, 54,668 women of reproductive age). We used data between January 2010 and June 2011 from a key informant surveillance system that recorded all births, deaths and stillbirths. Short birth interval was defined as an interval between consecutive births of less than 33 months. Initially, risk factors of a short birth interval were determined using a multivariate mixed effects logistic regression model. Independent risk factors were selected using *a priori* knowledge from literature review. An adjusted mixed effects logistic regression model was then used to determine the effect of up to 21-, 21-32-, 33-44- and 45-month and higher birth-to-birth intervals on pregnancy outcomes controlling for confounders selected through a directed acyclic graph.

**Results:**

We analysed 5,571 second or higher order deliveries. Average birth interval was 55 months and 1368/5571 women (24.6%) had a short birth interval (<33 months). Younger women (AOR 1.11 95% CI 1.08-1.15 per year increase in age), women who started their reproductive life later (AOR 0.95, 0.92-0.98 per year) and those who achieve higher order parities were less likely to experience short birth intervals (AOR 0.28, 0.19-0.41 parity 4 compared to 1). Women who were socioeconomically disadvantaged were more likely to experience a short birth interval (AOR 1.42, 1.22-1.65) and a previous adverse outcome was an important determinant of interval (AOR 2.10, 1.83-2.40). Very short birth intervals of less than 21 months were associated with increased stillbirth rate (AOR 2.13, 95% CI 1.28-3.53) and neonatal mortality (AOR 2.28 95% CI 1.28-4.05).

**Conclusions:**

Birth spacing remains a reproductive health problem in Bangladesh. Disadvantaged women are more likely to experience short birth intervals and to have increased perinatal deaths. Research into causal pathways and strategies to improve spacing between pregnancies should be intensified.

**Electronic supplementary material:**

The online version of this article (doi:10.1186/s12884-014-0427-6) contains supplementary material, which is available to authorized users.

## Background

Short birth intervals, defined as the time between two births, are known to have negative effects on maternal, perinatal and neonatal outcomes as well as on child health, though the precise mechanisms are poorly understood [1]. Short intervals are associated with increased preterm birth, low birth weight, and small for gestational age births, as well as perinatal death [2,3]. Neonatal and infant mortality is also higher after a negative outcome of the previous delivery, which is mediated primarily through short birth interval [4,5].

Neonatal and infant mortality is highest for birth to pregnancy intervals of 18 months and is slightly increased for birth to pregnancy intervals of 18–27 months [6]. The most recent WHO recommendation for a healthy pregnancy interval is at least two years (24 months), which corresponds to a birth-to-birth interval of 33 months under the assumption of nine months gestation [7]. The Government of Bangladesh recommends an interval of three years between the last live birth and the next attempt to get pregnant, which corresponds to a birth-to-birth interval of 45 months under the assumption of nine months gestation [8].

Corresponding with increasing levels of contraceptive use over the past 30 years (from 7 per cent in 1975 to 54 per cent by 2000) [9], Bangladesh has witnessed large reductions in fertility [9] and marked increases in median birth interval, increasing from 35 months in 1993–1994 to 47 months in 2011 [10]. However, more than two-thirds of women in Bangladesh are married by the age of 18 and the median age at first birth is approximately 18 years [10]. These young women have birth intervals of just 27 months and large differences are seen between socio-economic groups, with poorer, less educated women having shorter intervals [10-13]. The length of the birth interval is closely associated with the survival of the previous sibling [4,6]. The Bangladesh Demographic and Health Survey (BDHS) 2011 identifies a median birth interval 15 months shorter for children whose previous sibling died than for children whose previous sibling is alive (26 and 41 months, respectively) [10].

Mother’s age at first birth, parity, previous birth interval, mother’s working status, gender composition of the living children, and mass media are also described as determinants of birth intervals [11-13].

National strategies focused on family planning, reproductive health and safe motherhood have been well described [8] and are likely to represent a dynamic reproductive health context in Bangladesh that warrants contemporary description. Our study seeks to do this by not only describing birth spacing and its consequences on pregnancy outcomes, but also exploring the socioeconomic, demographic and reproductive factors that are associated with short birth intervals using prospective surveillance data from three districts in rural Bangladesh. Our findings are discussed in terms of opportunities for intervention strategies to prevent short birth intervals and the risks associated with them.

## Methods

### Study population

The study was conducted in nine unions (the lowest administrative level in Bangladesh) in three districts of Bangladesh – Bogra, Moulavibazar and Faridpur. The districts and unions represent part of a cluster randomised controlled trial of a community intervention to improve maternal and neonatal health described in detail elsewhere [14-16]. Only the control unions were included in the current descriptive study given that the aforementioned intervention could have influenced birth spacing or its consequences in intervention areas. The total population size of the nine unions was 282,643, representing 54,668 women of reproductive age residing in approximately 63,000 households. The study areas include tea-garden estates, which differ from much of non-tea garden areas in Bangladesh in terms of socioeconomics, access to services and history and are generally more disadvantaged than other areas [17,18].

### Data collection

Since 2004, a key informant surveillance system has been used in the study areas to record all births, neonatal deaths, and deaths of women of reproductive age [19]. 500 traditional birth attendants act as key informants, each monitoring a population of approximately 200 households or 1000 individuals. Most births in the study area are attended by traditional birth attendants, thus these women are aware of most if not all births in their community and are well placed to record pregnancy outcomes. Key informants are visited fortnightly by a monitor who collates data on births, either live or stillbirth, and neonatal and pregnancy-related deaths. 6–52 weeks after a registered event (birth or death), paid monitors visit women in their home to conduct a structured interview to gather data on socio-economic characteristics of the mother and her household, reproductive history, including birth interval, and history of previous pregnancy outcomes, as well as pregnancy, birth and postpartum experiences. Precise gestation cannot be reliably measured through these survey methods and so stillbirths are classified as such on the basis of reports of the birth of a child with no movement or other signs of life during the structured interview; it is unlikely that women’s reports or our survey tool would misclassify foetal deaths before 28 weeks gestation as stillbirths. All data are checked at the district office for quality and referred back to the field if incomplete or inaccurate. A random 10-20% sample of the respondents are revisited by supervisors to verify data quality. Data are sent to Dhaka for entry in an MS Access database, where further quality checks take place.

The current analysis was restricted to births taking place between January 2010 and June 2011. Observations were included if data on birth interval, pregnancy outcome and other predictors were complete.

### Definitions

We defined adverse pregnancy outcomes as the total number of stillbirths and neonatal deaths per 1,000 births. Perinatal mortality is comprised of the sum of stillbirths and deaths in the first week of life per 1,000 births. The neonatal mortality rate is presented as the number of newborn infants dying before reaching 28 days of age, per 1,000 live births and the early neonatal mortality rate as the number dying before reaching 7 days of age, per 1,000 live births.

Age was defined as the age of the mother at the time of the previous birth. Parity was defined as the number of times that a woman has given birth, regardless of whether the child was born alive or not. Maternal religion was categorised as either Muslim or other religion which included Hinduism and Christianity. The number of household assets was counted using a standard list of household items that included electricity, radio/tape recorder, fan, TV, fridge, phone, generator and bicycle and then dichotomised as ‘up to 3’ or ‘4 or more’ assets. Educational attainment was classified as either none/ primary only or one or more years in secondary or higher education.

Our data did not include the outcome of the immediately preceding pregnancy but did include the total number of pregnancies, miscarriages or abortions, stillbirths and live births for every mother as reported at the time of interview. These data were used to identify previous adverse pregnancy outcomes defined as a miscarriage or abortion, stillbirth or neonatal death resulting from any previous pregnancy.

### Statistical methods

Potential determinants of a short birth interval were chosen for inclusion in the statistical model and were arrived at a priori through a review of the literature. These included maternal age, parity, age at first pregnancy, maternal religion, education, household assets, tea garden residence and a history of a previous adverse pregnancy outcome. In examining the determinants of short birth interval we defined the outcome as an interval between consecutive births of any outcome (live or stillborn) of less than 33 months, corresponding to World Health Organisation recommendations. Initially, a univariate analysis was performed to determine the associations between individual predictors and short birth interval. A multivariate mixed effects logistic regression model, including all potential determinants of a short birth interval was then used to arrive at individually adjusted estimates. We assessed multicollinearity by calculating variance inflation factors (VIF). The VIF for parity was 3.60 and for age was 3.89, which indicates some, but acceptable collinearity. Given that we were interested in their joint effect, we kept both in the model. A likelihood ratio statistic was used to determine whether to include age and parity as a continuous or a categorical variable. Results indicate it was more appropriate to treat age as a discrete variable and parity as a categorical variable. The Receiver Operating Characteristic (ROC) was used to ascertain the predictive quality of the multivariate model.

Next, we evaluated short birth intervals as a determinant of an adverse pregnancy outcome. For this, birth interval was categorized as an interval between consecutive births of any outcome (live or stillborn) of <21 months, 21–32 months, 33–44 months and 45 months or longer, corresponding to birth to pregnancy intervals of less than one year, 1–2 years, 2–3 years and 3 or more years under the assumption of 9 months gestation.

We identified potential confounders based on a review of the literature. A Directed Acyclic Graph (DAG) was then used to assist in the selection of appropriate confounders by modelling the relationships between potential confounders, short birth interval and stillbirth or neonatal death [see Additional file 1]. The DAG was created using a pre-determined selection criteria in order to minimize biases that have recently been shown to be present when using traditional methods of confounder selection [20]. The final confounders used to model the effects of birth interval on birth outcome were maternal age, parity, education, religion, household assets, tea garden residence and previous adverse pregnancy outcome. A mixed effects logistic regression model was used to evaluate the association between birth interval categories and adverse outcomes of pregnancy adjusting for confounders and the clustered design of the study.

The DAG was drawn in Dagitty, an online tool [21]. All analysis were conducted using Stata version 12.1 (StataCorp).

### Ethics

This study uses data gathered as part of a larger cluster randomised trial of a community intervention. Community consent was acquired for intervention implementation and all monitoring activities. Individual informed consent was obtained before each interview. The current analyses were not subject to specific ethical review as they fall within the scope of the trial and monitoring activities that were approved by the University College London research ethics committee (identification number 1488/001) and the ethical review committee of the Diabetic Association of Bangladesh.

## Results

In the period January 2010-June 2011, 9,797 deliveries were registered with 5,911 second or higher order deliveries. The average birth interval was 4 years and 7 months (55 months). The average age of first pregnancy was 18.4 years and the average age of delivery 26.4 years (Table 1).

**Table 1:** **Crude and adjusted model of determinants of short birth interval defined as birth-to-birth interval <33 months**

	**Recommended birth interval (at least 33 months)**		**Short birth interval < 33 months**		**Crude OR**	**95% CI**		**OR** ^**†**^	**95% CI**	
**Age at onset of birth interval**	22.0 year^$^		22.8 year^$^		1.04	1.03	1.06	1.11	1.08	1.15
**Parity at onset of birth interval**										
1	2,811	50.5%	708	51.8%	*reference*			*reference*		
2	1,470	26.4%	329	24.0%	0.86	0.74	0.99	0.53	0.44	0.63
3	708	12.7%	168	12.3%	0.92	0.76	1.12	0.38	0.29	0.51
4+	582	10.4%	163	11.9%	1.16	0.95	1.41	0.28	0.19	0.41
**Age at first pregnancy**	18.3 year^$^		18.6 year^$^		1.04	1.02	1.07	0.95	0.92	0.98
**Adverse outcome of any previous pregnancy**										
Yes	2,021	36.3%	651	47.6%	2.28	1.95	2.67	2.10	1.83	2.40
No	3,550	63.7%	717	52.4%	*reference*			*reference*		
**Religion**										
Muslim (%)	4,605	82.6%	1,152	84.2%	*reference*			*reference*		
Other (%)	966	17.3%	216	15.8%	0.86	0.73	1.02	0.68	0.53	0.87
**Education**										
None or primary education only	3,408	61.2%	810	59.2%	*reference*			*reference*		
Secondary or above	2,163	38.8%	558	40.8%	1.11	0.98	1.26	1.26	1.09	1.45
**Household assets**										
0-3	4,043	72.7%	1040	76.0%	1.22	1.06	1.41	1.42	1.22	1.65
4+	1,520	27.3%	328	24.0%	*reference*			*reference*		
**Tea garden resident**										
Yes	663	11.9%	169	12.4%	1.06	0.88	1.28	1.41	1.07	1.87
No	4809	88.1%	1.199	87.6%	*reference*			*reference*		

^†^Adjustment for all other variables. ^$^Average.

We checked for data completeness and 340 observations (5.8%) had missing data. 330 observations had a missing birth interval and 10 observations missed other covariates (parity, education). Neonatal mortality was higher among those cases with missing data and younger age, lower parity, previous adverse outcomes, Christian or Hindu religion and higher education groups were overrepresented [see Additional file 2]. A complete case analysis was performed, using 5,571 pregnancies without any missing data.

### Determinants of birth interval <33 months

1,368 women (24.6%) had a birth interval shorter than 33 months. The determinants of short birth intervals are described in detail in Table 1. Results from the mixed effects multivariate analysis indicate that the risk of having a short interval increases with age. The likelihood of a short birth interval increased by 11% with each additional year of age (adjusted odds ratio (AOR) 1.11 95% confidence interval (CI) 1.08-1.15). Women who delivered a third or higher order baby were less likely to xperience a short birth interval compared to women who delivered their second baby. Parity of four or more was associated with 72% decrease in the odds of a short birth interval compared to a parity of one at the start of the birth interval (AOR 0.28, 0.19-0.41). The likelihood of a short birth interval decreased 5% for every year that a woman’s first pregnancy was delayed (AOR 0.95, 0.92-0.98). Women who experienced a previous adverse outcome of pregnancy were twice as likely to have a birth interval of less than 33 months compared to those women whose babies survived the neonatal period (AOR 2.10, 1.83-2.40). Christian or Hindu women were 32% less likely to experience a short birth interval than Muslim women (AOR 0.68, 0.53-0.87). Women with secondary education or higher had a 26% increased likelihood of a short birth interval (AOR 1.26, 1.09-1.45) whilst women from a household with up to 3 assets were 42% more likely to have a short interval compared to those with four or more assets (AOR 1.42, 1.22-1.65). Tea-garden residence was associated with a 41% increase in the odds of a short birth interval (AOR 1.41, 1.07-1.87). The ROC showed that the model was a reasonable fit (0.63).

### Short birth interval and adverse outcome of pregnancy

Compared to birth intervals of 45 months or longer, birth intervals of less than 21 months were associated with a greater than two-fold increased risk of adverse pregnancy outcome (AOR 2.23 95% CI 1.51-3.29), as well as increased risk of perinatal mortality (AOR 2.33, 95% CI 1.55-3.50), stillbirth (AOR 2.13, 95% CI 1.28-3.53), neonatal mortality (AOR 2.28 95% CI 1.28-4.05) (Table 2). There was no evidence of increased risk of adverse pregnancy outcomes associated with birth intervals of 21–32 months and 33–44 compared to longer intervals.

**Table 2:** **Exposure models of adverse outcomes of pregnancy by birth interval**

**Birth interval (months)**	**N**	**Events**		Crude OR*	**95% CI**		**Adjusted OR** ^**†**^	**95% CI**	
		**N**	**/1,000**						
**Adverse outcome of pregnancy**									
>45	3,173	143	45.1	*reference*			*reference*		
33-44	1,030	54	52.4	1.19	0.86	1.65	1.21	0.87	1.70
21-32	879	42	47.8	1.07	0.75	1.52	1.13	0.78	1.65
<21	489	44	90.0	*2.12*	1.49	3.03	*2.23*	1.51	3.29
**Perinatal mortality**									
>45	3,173	124	39.1	*reference*			*reference*		
33-44	1,030	50	48.5	1.29	0.92	1.82	1.31	0.92	1.87
21-32	879	37	42.1	1.09	0.75	1.59	1.15	0.77	1.71
<21	489	41	83.8	*2.31*	*1.60*	*3.35*	*2.33*	1.55	3.50
**Still birth rate**									
>45	3,173	79	24.9	*reference*			*reference*		
33-44	1,030	28	27.2	1.09	0.70	1.69	1.10	0.70	1.73
21-32	879	19	21.6	0.85	0.51	1.42	0.92	0.54	1.56
<21	489	25	51.1	2.10	1.32	3.33	2.13	1.28	3.53
**Neonatal mortality**									
>45	3094	64	20.7	*reference*			*reference*		
33-44	1002	26	25.9	1.31	0.82	2.09	1.35	0.83	2.20
21-32	860	23	26.7	1.32	0.82	2.16	1.38	0.83	2.32
<21	464	19	40.9	2.10	1.24	3.55	2.28	1.28	4.05
**Early neonatal mortality**									
>45	3094	45	14.5	*reference*			*reference*		
33-44	1002	22	22.0	1.66	0.98	2.81	1.68	0.65	1.84
21-32	860	18	20.9	1.51	0.87	2.65	1.54	0.55	2.23
<21	464	16	34.5	2.63	1.46	4.74	2.59	0.53	2.73

*The crude analyses were adjusted for clustering.

^†^The OR’s were adjusted for maternal age, parity, previous birth outcome, maternal religion, education and household assets, tea garden residence and clustering.

## Discussion

Using data from a large population based surveillance system from three districts in rural Bangladesh we identified demographic and reproductive predictors of short birth interval and identified important consequences of short intervals on pregnancy outcomes. Younger women, women who start their reproductive life later in life and those who achieve higher order parities are less likely to experience short birth intervals. A previous adverse outcome is an important determinant of short birth interval, which in turn increases the likelihood of subsequent adverse outcomes including stillbirth, perinatal and neonatal death, potentially creating a detrimental cycle of increased risk to reproductive, maternal and newbron health, particularly amongst the most socioeconomically disadvantaged groups.

Between the early 1950s and the early 2000s, the total fertility rate in Asia dropped from 5.7 to 2.4 births per woman [22]. This resulted in reduced family size and increases in average birth intervals and may in part be explained by the increase in contraceptive use – from 7 per cent in 1975 to 54 per cent by 2000 in Bangladesh [9]. Although the reduction in fertility rate has slowed, there is still a downward trend. Data limitations in the current analysis prevented us from examining the role of family planning, breastfeeding and fertility trends in our study setting, but our finding that young women are less likely than older women to experience short birth intervals suggests an overall shift in reproductive choices that may reflect these wider changes in family planning and fertility observed across the continent.

The current total fertility rate in Bangladesh is estimated as 2.3 children and average ideal family size is 2.2 children [10]. The observation that women who have achieved a parity of two or more and pursue a subsequent pregnancy are less likely to have a short birth interval than women with parity of one may not be surprising therefore. These women may have achieved their desired family size and may feel less pressure or be in less of a hurry to get pregnant again. However, it is difficult in our analysis to disentangle the effects of age, parity and age at first pregnancy, which will all correlate with average past birth intervals and are associated with the interval of interest. Nevertheless, including each of these parameters in our adjusted model allows some estimation of the independent effect of each.

We observe that women with a higher educational attainment have an increased risk of short birth intervals, which is surprising. Data from the DHS from Bangladesh as well as other countries suggest better educated women have longer birth intervals [10,23]. A hypothesis could be that more educated women in our study may have married later in life and subsequently hurried to establish a family. However, the relatively small difference in age at first pregnancy between education groups observed in our data is relatively small (18.27 compared to 18.63 years, p < 0.01) and does not support this. A further hypothesis is that better educated women may wish to compress childbearing into fewer years and participate in non-childbearing activities, but further quantitative and qualitative research is required to investigate this.

We have shown that a previous adverse outcome is a determinant of short birth interval and, in a separate analysis, that a short birth interval is a risk factor for an adverse pregnancy outcome. A limitation of our study is that we are unable to disentangle the possible “scarring” effect of a previous birth interval and unobserved family characteristics that cause both clustering of perinatal deaths and short birth intervals (family heterogeneity). A previous adverse outcome may have a “scarring” effect, because it causes women to rush into a next pregnancy (replacement) without properly recovering from the previous pregnancy. Women who experience a short birth interval may be subject to other factors that could result in clustering of adverse pregnancy outcomes, such as reduced access to health facilities which could reduce both access to family planning as well as delivery and perinatal care. A study by Saha *et al.,* using advanced panel data analysis techniques and longitudinal data from Matlab, Bangladesh, demonstrates that a short birth interval is the most important pathway to explain neonatal death of the consequent child after a previous adverse neonatal death adjusted for unobserved family heterogeneity [4].

Our observations confirm that the risk of a short birth interval is highest for those least well-off, both in terms of household assets and tea garden residence. Analysis of BDHS data also indicates that birth intervals are shortest and the difference between desired spacing and actual birth interval is biggest for the least well-off [23]. Differences in the use of family planning methods could explain the differences in birth intervals between socioeconomic groups. Rahman *et al.* demonstrated that replacement level fertility has almost been achieved in women who are better educated and socioeconomically better off, while replacement level fertility has not been achieved yet for the less well-off [9].

Very short birth-to-pregnancy intervals of 18 months or shorter are associated with elevated risk of infant, neonatal and perinatal mortality, low birth weight, small size for gestational age, and pre-term delivery [2-6]. A birth-to-pregnancy interval of 18–27 months may pose some “residual” risk [2]. We observe that a very short birth interval less than 21 months (birth-to-pregnancy of less than 12 months when pregnancy is carried to term) is associated with an increased risk of adverse pregnancy outcomes, but intervals of 24–32 months (birth-to-pregnancy interval of 12–23 months when pregnancy is carried to term) and 33–44 months (birth-to-pregnancy interval of 24–35 months) do not appear to be. Based on these findings, the World Health Organisation’s recommendation to wait two years after a live birth before attempting a next pregnancy, and the Government of Bangladesh’s recommended birth-to-pregnancy interval of 3 years, may be overly cautious as far as perinatal outcomes are concerned.

The purpose of observational studies is to inform intervention, policy and practice. Our findings suggest that women from poor socioeconomic backgrounds and women who experienced an adverse previous pregnancy should receive particular attention from family planning programs in rural Bangladesh. Post-partum counselling including family planning counselling for bereaved mothers who experienced an adverse outcome is likely to be important in addressing short birth intervals and their consequences. An approach that may have potential in targeting women from poor socioeconomic backgrounds is community mobilisation through women’s groups on reproductive and women’s health. The approach has been shown to be effective at improving maternal and neonatal health, especially for the most marginalised women [15,24,25].

## Conclusion

Birth spacing remains an issue that deserves urgent attention in Bangladesh. Disadvantaged women are more likely to experience short birth intervals and suffer their consequences. Further research should look into causal pathways as well as strategies to improve spacing between pregnancies whilst efforts to prevent adverse pregnancy outcomes, an important determinant of birth spacing, should be intensified.

## Additional files

Additional file 1: Figure. The Directed Acyclic Graph (DAG) outlines the causal assumptions of short birth interval as a determinant of an adverse pregnancy outcome. Given these causal assumptions, the analysis is adjusted for all variables that can potentially cause bias (white circles) using the backdoor criterion. Additional file 2: Table. Comparison of pregnancies with missing and complete data.
