# Supplementary figures and images for: Determinants and consequences of short birth interval in rural Bangladesh: a cross-sectional study
Source: BMC Pregnancy Childbirth. 2014 Dec 24;14:427. doi: 10.1186/s12884-014-0427-6 (PMC4314752; doi:10.1186/s12884-014-0427-6)

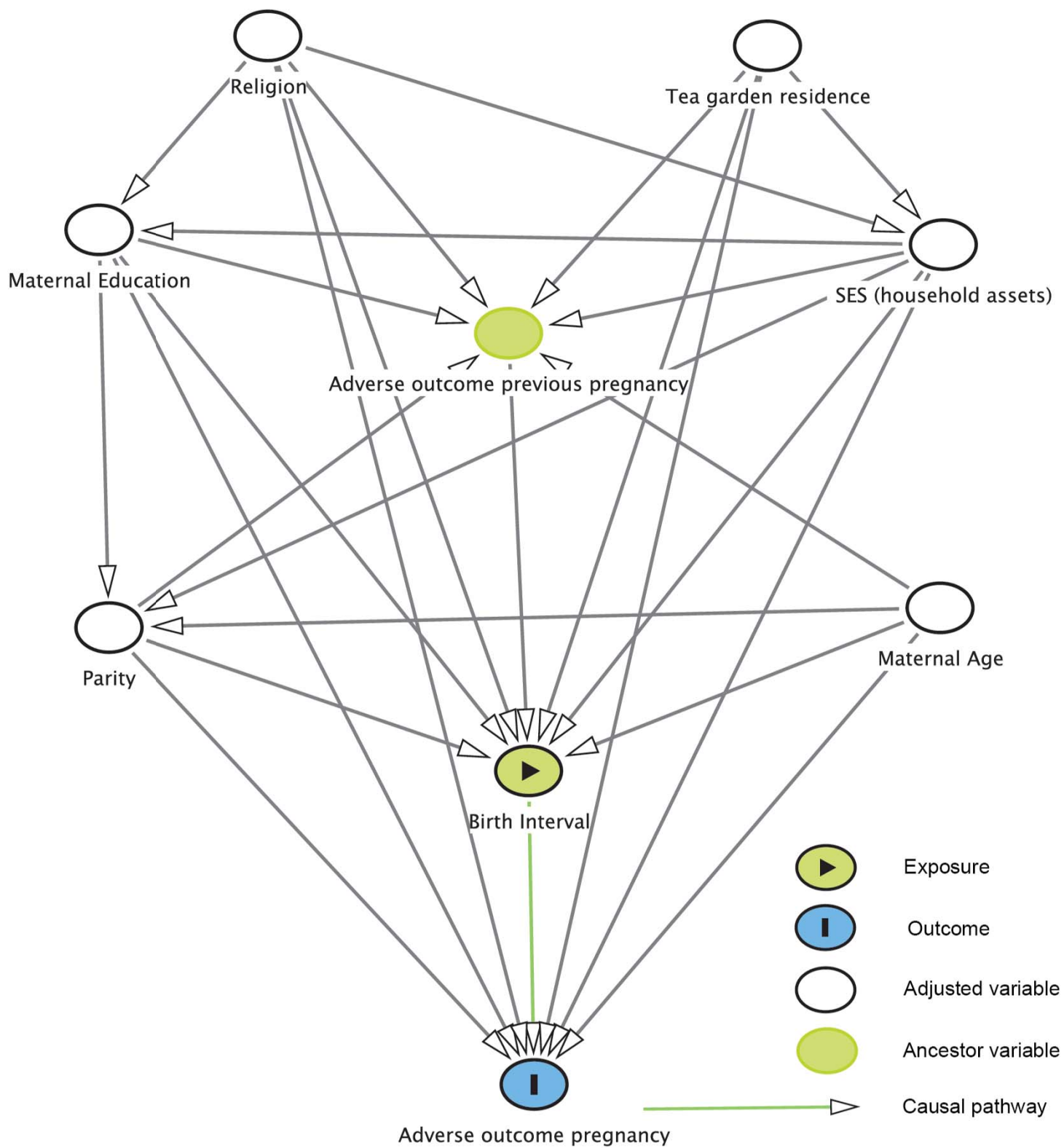

Supplement: Additional file 1: Figure. — The Directed Acyclic Graph (DAG) outlines the causal assumptions of short birth interval as a determinant of an adverse pregnancy outcome. Given these causal assumptions, the analysis is adjusted for all variables that can potentially cause bias (white circles) using the backdoor criterion. [file 12884_2014_427_MOESM1_ESM.pdf]
