# Supplementary material for: Determinants and consequences of short birth interval in rural Bangladesh: a cross-sectional study
Source: BMC Pregnancy Childbirth. 2014 Dec 24;14:427. doi: 10.1186/s12884-014-0427-6 (PMC4314752; doi:10.1186/s12884-014-0427-6)
Supplement: Additional file 2: Table. — Comparison of pregnancies with missing and complete data. [file 12884_2014_427_MOESM2_ESM.pdf]

Table - Comparison of pregnancies with missing and complete data.

	Observations with complete data (n=5571)	Observations with missing data (n=340)	Δ complete-missing P value*
% Adverse birth outcome	5.1%	7.1%	0.110
% Neonatal death**	2.4%	4.2%	0.043
% Still birth	2.7%	2.9%	0.800
Maternal age			0.000
35+	7.7%	0.9%	
25-34	58.6%	19.7%	
<25	33.7%	79.4%	
Gravidity			0.000
2	43.9%	81.5%	
3	27.9%	12.9%	
4	14.5%	3.2%	
5+	13.7%	2.4%	
No assets			0.000
None	27.3%	38.8%	
1-3	49.4%	42.9%	
4+	23.3%	18.2%	
Previous adverse outcome	36.3%	90.8%	0.000
Tea garden	11.9%	14.7%	0.123
% Muslim	82.7%	77.2%	0.000
% Secondary education or higher	38.8%	60.7%	0.000

* using Pearson's chi square
** pregnancies ending in still birth excluded. Observations with complete data n=5420. Observations with missing data n=330
